# Wenshen Shengjing Decoction Improves Early Embryo Development by Maintaining Low H3K27me3 Levels in Sperm and Pronuclear Embryos of Spermatogenesis Impaired Mice

**DOI:** 10.1155/2021/8035997

**Published:** 2021-09-27

**Authors:** Yanmei Sun, Fan Gao, Da Xu, Lei Lu, Qianggen Chen, Zheqing Yang, Xuenan Wang, Xiaoyan Pan

**Affiliations:** ^1^Center for Reproductive Medicine, Jilin Medical University, Jilin 132013, China; ^2^Department of Neonatology, Jilin Central General Hospital, Jilin 132011, China; ^3^Reproductive Medicine Center of the Affiliated Hospital of Jining Medical College, Jining 272029, China

## Abstract

Many ingredients in Wenshen Shengjing Decoction (WSSJD) can cause epigenetic changes in the development of different types of cells. It is not yet known whether they can cause epigenetic changes in sperms or early embryos. Here, we investigated the role of WSSJD in epigenetic modifications of sperms or early embryos and early embryo development. A mouse model with spermatogenesis disorders was established with cyclophosphamide (CPA). WSSJD was administrated for 30 days. The male model mice after the treatment were mated with the female mice treated with superovulation. The embryo development rate of each stage was calculated. Immunofluorescence staining was used to detect the expression of H3K27me3 in sperm, pronuclear embryos, and 2-cell embryos. Western blotting was used to detect the expression of histone demethylase KDM6A and methyltransferase EZH2 in 2-cell embryos with developmental arrest. The expressions of zygotic genome activation genes (*ZSCAN4*, *E1F1AX*, *HSPA1A*, *ERV4-2*, and *MYC*) in 2-cell embryos with developmental arrest were analyzed with qRT-PCR. Comparing with the control group, CPA destroyed the development of seminiferous epithelium, significantly increased the expression level of H3K27me3 in sperm, reduced the expression ratio of H3K27me3 in female and male pronuclei, delayed the development of 2-cell embryos, and increased the developmental arrest rate and degeneration rate of 2-cell embryos. Moreover, the expressions of EZH2 and H3K27me3 were significantly increased in the 2-cell embryos with developmental arrest, and the expression of zygotic genome activation genes (*ZSCAN4*, *E1F1AX*, *HSPA1A*, *ERV4-2*, and *MYC*) was significantly decreased. Compared with the CPA group, WSSJD promoted the development of seminiferous epithelium, maintained a low level of H3K27me3 modification in sperm and male pronucleus, significantly increased the development rate of 2-cell embryos and 3-4 cell embryos, and reduced the developmental arrest rate and degeneration rate of 2-cell embryos. WSSJD may promote early embryonic development by maintaining a low level of H3K27me3 modification in sperm and male pronucleus and regulating the zygotic genome activation in mice with spermatogenesis disorders induced by CPA.

## 1. Introduction

Wenshen Shengjing Decoction (WSSJD) is a traditional Chinese compound medicine, which is widely used to treat male spermatogenesis disorders [1–5]. WSSJD is composed of 15 kinds of Chinese herbs, mainly including Ginseng, velvet antler, Cynomorium, Astragalus, Epimedium, and Angelica [1]. It can maintain high androgen concentration in testicular tissue, promote the development of spermatogenic cells [1], inhibit the apoptosis of spermatogenic cells [5], and increase the production and maturity rate of sperms [2]. An important criterion for the efficacy of drugs for male spermatogenesis disorders is normal fertility. Therefore, the efficacy of WSSJD depends on its effect on early embryonic development.

Epigenetic modification is an important method of gene expression regulation, which can regulate DNA replication, transcription, and repair [6], and plays an important regulatory role in the development of sperms and early embryos. It has been found that many effective ingredients in WSSDJ can participate in the regulation of cell epigenetic modification, such as ginsenosides, astragalus polysaccharides, and limoxine [7–10]. Ginsenoside Rg3 can regulate the acetylation modification of H3K14/K9 and H4 K12/K5/K16 in ovarian cancer cells [7]. Astragalus polysaccharide can regulate the methylation status of DNA in the colonic epithelium [8]. Tetramethylpyrazine can cause histone H3 and H4 acetylation modification in neural stem cells [9], which are closely related to the changes in the modification pattern of H3K27me3 in mouse cardiomyocytes [10]. Our previous research found that WSSJD regulated the modification level of K3K9me2 in spermatocytes and inhibited spermatocyte apoptosis [5]. Therefore, it is speculated that WSSJD may also be involved in the regulation of epigenetic modification of sperm or early embryos.

H3K27me3 plays an important regulatory role in the development of sperm and early embryos [11–16], and it is involved in the regulation of gene transcription inhibition [10]. H3K27me3 is widely present in testicular tissues [11]. Polycomb protein SCML2 promotes the modification of spermatogonia H3K27me3 by binding to the promoter region of undifferentiated spermatogonia [12]. H3K27me3 regulates the expression of meiosis-related genes and involves the meiosis of spermatocytes [13]. Moreover, H3K27me3 is still retained in mature sperm of mice, humans, and zebrafish [14–16]. H3K27me3 is genetically inherited in mammals [14–16], and the inhibitory histone marker H3K27me3 in drosophila embryos could be passed to their offspring [17]. H3K27me3 in early embryos can accumulate in the regulatory region of H3K27ac to prevent premature activation of zygotic genome activation genes [17].

Cyclophosphamide (CPA) is a commonly used antitumor and immunosuppressive drug. Its metabolites can bind to DNA and proteins in cells, activate enzymes, and cause cell death [18]. The testis containing the dividing spermatogenic cells at various levels is particularly sensitive to CPA. It is reported that CPA had a high toxic effect on male reproduction and could cause oligospermia and azoospermia [19]. CPA is often used for the establishment of animal models with spermatogenesis disorders [20, 21].

Considering that WSSJD may be involved in the epigenetic regulation of sperms and embryos and that H3K27me3 is a marker involved in the regulation of sperms and early embryo development [11–16], therefore, we aim to investigate the possible effect of WSSJD on the modification of H3K27me3 in sperms or early embryos and early embryo development. The mouse model with spermatogenesis disorders was induced with CPA. The effects of WSSJD on early embryo development and H3K27me3 modification were analyzed. Our findings may provide experimental evidence for demonstrating the therapeutic mechanism of WSSJD on spermatogenesis disorders.

## 2. Materials and Methods

### 2.1. Animals

A total of 180 male Kunming mice (10-weeks-old; 35–40 g) and 80 female Kunming mice (8-weeks-old; 25–30 g) were provided by the Changchun Institute of Biological Products Co., Ltd. (Changchun, China). The present study was conducted with approval from the Ethics Committee of Jilin Medical University (Jilin, China). Mice were housed at 23 ± 3°C room temperature, with 55–65% relative humidity, and under a 12 h dark-light cycle. Mice were given free access to a standard laboratory mouse diet and sterile water.

### 2.2. Preparation of WSSJD

The 15 Chinese herbs of WSSJD, including *Panax ginseng*, 6 g; *Cynomorium songaricum*, 9 g; Radix Astragali, 12 g; *Epimedium brevicornum*, 6 g; Cornu Cervi Nippon Parvum, 1 g; *Cistanche deserticola*, 9 g; *Angelica sinensis*, 6 g; Flatstem Milkvetch Seed, 9 g; Rhizoma Dioscoreae, 15 g; Largehead Atractylodes Rhizome, 6 g; *Ligusticum wallichii*, 3 g; Radix Paeoniae Alba, 6 g; *Cinnamomum cassia*, 1 g; Costustoot, 1.5 g, and Fructus Foeniculi, 3 g. [1], were purchased from Tongrentang (Beijing, China). These herbs were decocted according to the traditional method of Chinese medicinal decoction [22]. The decoction was subsequently heated at 80°C in a water bath for 6-7 h until the concentration reached 2 g crude drug/ml, and the decoction was stored at 4°C prior to use.

### 2.3. Animal Model and Treatment

All 180 mice were randomly divided into 3 groups (60 mice per group) of Control, CPA, and WSSJD groups. Mice in the CPA and WSSJD groups were intraperitoneally (i.p.) injected with 80 mg/kg/day CPA (Shanxi Powerdone Pharmaceutics Co., Ltd., Datong, China) for 5 days [23]. Then, mice in the WSSJD group were administered with 12 g crude drug/kg/day of WSSJD by gavage for 30 days. Mice in the control and CPA groups underwent a daily gavage with an equal volume of normal saline throughout the 30-day experimental period. At the end of the experimental period (at day 31), one-half amount of male mice were sacrificed by cervical dislocation. The testes were quickly removed. The epididymis was also quickly removed, punctured with a 26-gauge needle, and incubated at 37°C for 10 min to fully release the sperm from the epididymal tail. The other half of male mice were used to mate with the female mice with superovulation to test their fertility.

### 2.4. Hematoxylin and Eosin Staining

The testes were fixed with 4% paraformaldehyde, dehydrated by gradient ethanol, transparent in xylene, embedded in paraffin, made into 5 *μ*m sections, and stained with Hematoxylin and Eosin according to routine procedure. The histopathological changes in the seminiferous tubules were observed under a light microscope.

### 2.5. Immunofluorescence Staining of Sperms

Sperm suspensions from 5 mice of each group were diluted in distilled water, smeared on a cover glass, and dehydrated at room temperature for 2 h. Sperm deagglutination solution (25 mM DTT, 0.2% Triton X-100 and 200 IU/mL heparin) was added dropwise to the smear and incubated in a 37°C incubator for 15 min. The sperm deagglutination solution was discarded, and the 3.7% paraformaldehyde solution was used for fixation for 20 min. After washing with PBS and blocking with 5% BSA for 2 h at room temperature, the sample was incubated with rabbit-derived anti-H3K27me3 (A2363, ABclonal) primary antibody for 2 h. After washing three times with PBS, the goat anti-rabbit FITC-labeled secondary antibody (AS011, ABclonal) was added and incubated for 1 h. Hoechst 33342 (14533, Sigma) was used to stain the sperm nucleus. Finally, the sample was mounted with antifluorescence quenching mounting fluid (AR1109, BOSTER) and observed under the oil microscope of Olympus IX-53 with the microscopic image acquisition system (CellSens Dimension) (Olympus, Tokyo, Japan). Five sections were obtained from each mouse. Five high-power fields (×1000) were randomly selected, and ImageJ software (NIH) was used to analyze the average fluorescence intensity value of H3K27me3 in sperm.

### 2.6. Embryo Collection and Immunofluorescence Staining

Through observation of vaginal changes and vaginal cytology as described by Byers et al. [24], the estrous cycle of mice was determined. Proestrus mice were selected for follicular stimulation and mating process. Then, female mice were subjected to superovulation treatment of 10IU pregnant mare serum gonadotropin (i.p.) (cat# 200803; Ningbo Second Hormone Factory Co. Ltd., Ningbo, China), and 10IU human chorionic gonadotropin (HCG) (cat# 190703; Ningbo Second Hormone Factory Co. Ltd., Ningbo, China) injection 48 h later. After that, the female mice were caged with male mice at 1 : 1 ratio. The mouse oviducts were obtained at 21 h, 41-42 h, 43-44 h, 45-46 h, and 52 h after HCG injection. The pronuclear embryos, 2-cell embryos, and 3-4 cell embryos were collected from the ampulla of the oviducts. The 0.1% hyaluronidase (H3506, Sigma) was used to remove cumulus cells from the pronuclear embryos. The development rate of embryos at a specific stage was calculated as the number of embryos at that stage divided by the total number of embryos obtained from the oviducts.

The obtained pronuclear embryos and 2-cell embryos were fixed in 3.7% paraformaldehyde solution for 20 min and in 0.1% Trion X-100 in PBS solution for 15 min. After blocking with 5% BSA blocking solution for 1 h, the embryos were incubated with rabbit-derived anti-H3K27me3 (A2363, ABclonal) primary antibody for 2 h. After washing three times with PBS, the embryos were incubated with a goat anti-rabbit FITC-labeled secondary antibody (AS011, ABclonal) for 1 h. After staining with Hoechst 33342 (14533, Sigma), the embryos were mounted and observed by a laser scanning confocal microscope (Olympus, FV1000). ImageJ software was used to analyze the average fluorescence intensity of H3K27me3 on 10 embryos in each group.

### 2.7. Western Blotting

The 2-cell embryos (*n* = 60 each group) were subjected to protein lysis (950 *μ*L Laemmli sample buffer + 50 *μ*L *β*-mercaptoethanol + 0.5 *μ*L protease inhibitor). The proteins were collected after full lysis at room temperature. After separation by SDS-PAGE, the proteins were transferred to the PVDF membrane. The membrane was blocked with 5% skimmed milk for 1 h and then incubated with rabbit polyclonal antibody KDM6A (lysine-specific demethylase 6A) (A8159, Abclonal), rabbit polyclonal antibody EZH2 (Enhancer of zeste homolog2) (A16846, Abclonal), and rabbit polyclonal antibody Lamin A/C (A0249, Abclonal) at 4°C overnight. After washing with PBST, the membrane was incubated with goat anti-rabbit HRP-labeled secondary antibody (AS041, Abclonal) for 2 h at room temperature. Then, the enhanced chemiluminescence color development was performed. The membrane was scanned using ChemiDOC XRS + imaging systems (Bio-Rad Laboratories, Hercules, CA, USA). ImageJ image analysis software was used to analyze the relative expression levels of KDM6A and EZH2.

### 2.8. Quantitative Real-Time PCR (qRT-PCR)

RNAs were extracted from embryos (*n* = 60 each group) using Rneasy Micro Kit (Qiagen, Hilden, Germany) and transcribed into cDNA. Reverse transcription was performed in a 20 *μ*L reverse transcription system (1 *μ*L random primers, 1 *μ*L Oligo dT Primer, 4 *μ*L Reverse Transcription buffer, and 1 IU/mL PrimeScriptTEMRT Enzyme Mix I (TaKaRa, Dalian, China)). The mRNA levels of *HSPA1A* (recombinant heat shock 70 kDa protein 1A) , *MYC* (myelocytomatosis oncogene homolog), *EIF1AX* (eukaryotic translation initiation factor 1AX), *ERV4* (endogenous retroviral sequence 4), and *ZSCAN4* (zinc finger and SCAN domain containing 4) were measured with SYBR Premix Ex Taq (Takara, Dalian, China) on the iQ5 Multicolor Real-time PCR Detection System (Bio-RAD). The specific primers used are shown in Table 1. Real-time PCR reaction system included Premix Ex TaqTM II, forward/reverse primers and cDNA template. PCR reaction conditions were 95°C predenaturation for 30 seconds, 40 cycles of 95°C denaturation for 5 seconds, 60–62°C annealing for 20 seconds, and 72°C extension for 30 seconds. The housekeeping gene GAPDH was used as an internal reference. The 2^−△△Ct^ method was used to calculate the relative expression level of the target gene.

### 2.9. Statistical Analysis

The data were statistically analyzed by SPSS 17.0 software and were expressed as mean ± standard deviation (SD). One-way ANOVA (one-way ANOVA) and LSD post hoc test were used to compare differences of multiple groups. *P* < 0.05 was considered statistically significant.

## 3. Results

### 3.1. WSSJD Promotes the Development of Seminiferous Epithelium

**Figure 1:**
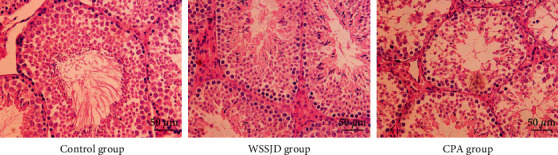
Histopathological changes in the seminiferous tubules.

Hematoxylin and Eosin staining was performed to assess the histopathological changes in the seminiferous tubules. As shown in Figure 1, the seminiferous tubules in the testes of the WSSJD group and the control group were relatively compact and intact with rich testicular interstitial tissue but without atrophy or collapse. The spermatogenic cells were tightly arranged, and the number of epithelial cell layers was more. In the CPA group, the seminiferous tubules were shrunken at the edges, with reduced diameter. The number of epithelial cell layers was reduced; the spermatogenic cells were arranged scattered, and mature sperm were rare in the lumen (Figure 1). Thus, WSSJD could promote the development of seminiferous epithelium.

### 3.2. WSSJD Maintains the Low Expression Level of H3K27me3 in Sperm

**Figure 2:**
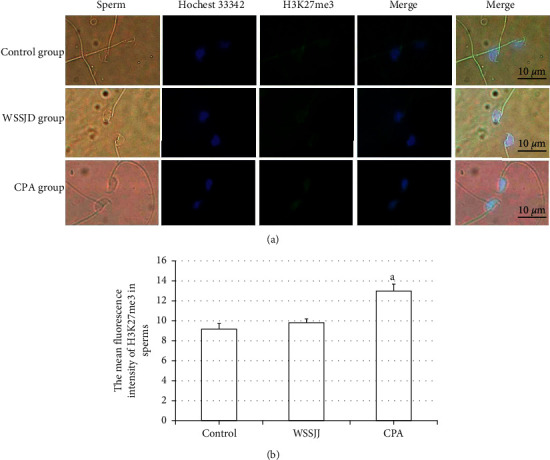
Expression of H3K27me3 in mouse sperm. (a) Immunofluorescence labeling of H3K27me3 in mouse sperm. H3K27me3 is marked in green, and the sperm nucleus is marked in blue. (b) The average fluorescence intensity of H3K27me3 in sperm (*N* = 25). Compared with the control group and the WSSJD group, ^a^*P* < 0.05.

Mature sperm were obtained from the epididymal tail of mice in the control group, WSSJD group, and CPA group, and the sperm was subjected to H3K27me3 immunofluorescence staining (Figure 2(a)). H3K27me3 was present in the nucleus of sperm (Figure 2(a)). There was no significant difference in terms of average fluorescence intensity of sperm H3K27me3 between the WSSJD group and the control group (Figure 2(b)), while the average fluorescence intensity of sperm H3K27me3 in the CPA group was significantly higher than that of the WSSJD group and the control group (*P* < 0.05, Figure 2(b)).

### 3.3. WSSJD Maintains Low H3K27me3 Modification Pattern in Male Pronuclear Embryos

**Figure 3:**
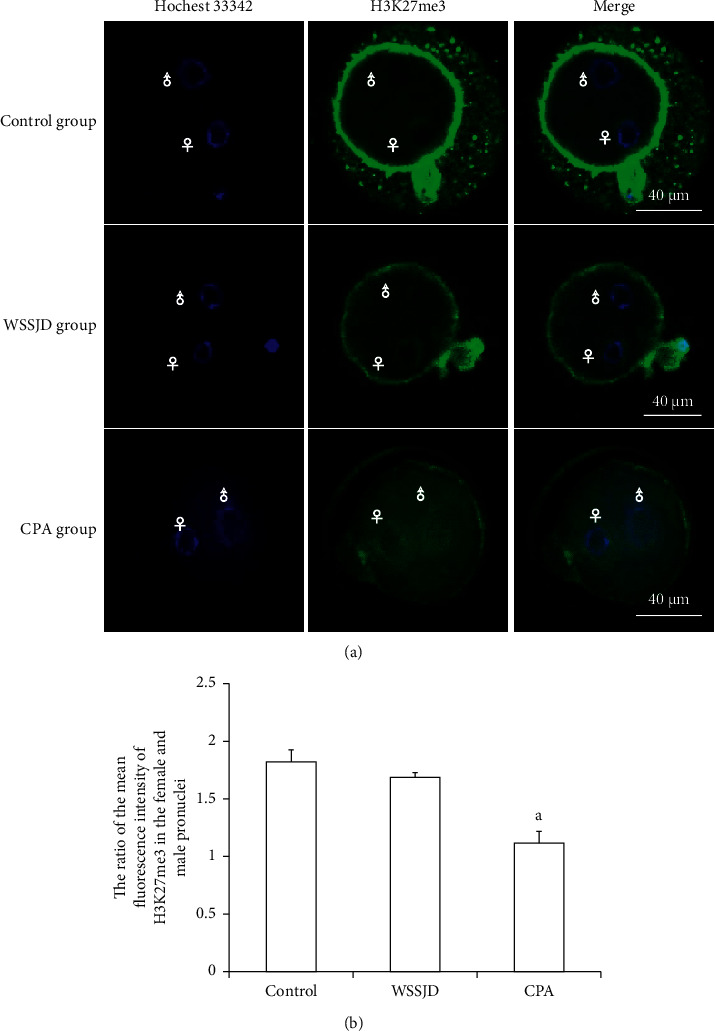
Expression of H3K27me3 in mouse PN2 and PN3 pronuclear embryos. (a) Immunofluorescence labeling of H3K27me3 in mouse pronuclear embryos. H3K27me3 is marked in green, and embryonic nuclei are marked in blue. Maternal pronucleus (♀) and paternal pronucleus (♂). (b) The ratio of the average fluorescence intensity of H3K27me3 in male and female pronuclei (*N* = 10). Compared with the control group and WSSJD group, ^a^*P* < 0.05.

Pronuclear embryos were collected from mouse oviduct 21 h after HCG injection (Figure 3(a)). According to the location and size of male and female pronuclei, the pronuclear embryos were divided into PN1-PN5 [25]. The male and female pronuclei of PN2 and PN3 pronuclear embryos are enlarged, and there is a certain distance between the male and female pronuclei. The larger one is the male pronucleus (♂), and the smaller one is the female pronucleus (♀). In this study, PN2 and PN3 pronuclear embryos were selected for H3K27me3 immunofluorescence staining (Figure 3(a)). In the WSSJD group and the control group, H3K27me3 was mainly expressed in the female pronucleus, and almost no H3K27me3 expression was seen in the male pronucleus. In the control group, H3K27me3 was not only expressed in the female pronucleus but also expressed in the male pronucleus. To further determine its expression level in male and female pronuclei, the average fluorescence intensity of H3K27me3 in male and female pronuclei was determined (Figure 3(b)). The ratio of the average fluorescence intensity of H3K27me3 in the female and male pronuclei of the WSSJD group was not significantly different from that of the control group but was significantly higher than that of the CPA group (*P* < 0.05).

### 3.4. WSSJD Promotes 2-Cell Embryo Development and Reduces Embryo Degeneration

**Figure 4:**
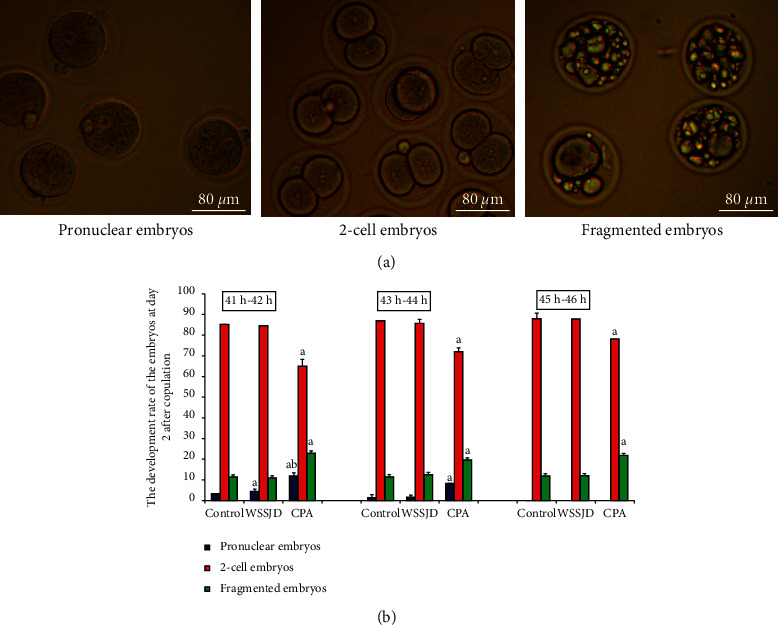
Embryos obtained from mouse oviduct at 41 h–46 h after hCG injection. (a) Embryos obtained from the oviduct of mice in the control group at 44 h after hCG injection, including pronuclear embryos, 2-cell embryos, and fragmented embryos. (b) The ratio of embryos of each stage collected at 41 h-42 h, 43 h-44 h, and 45 h-46 h after hCG injection in different experimental groups (*N* = 10) was calculated. Compared with the control group, ^a^*P* < 0.05; compared with the WSSJD group, ^b^*P* < 0.05.

**Figure 5:**
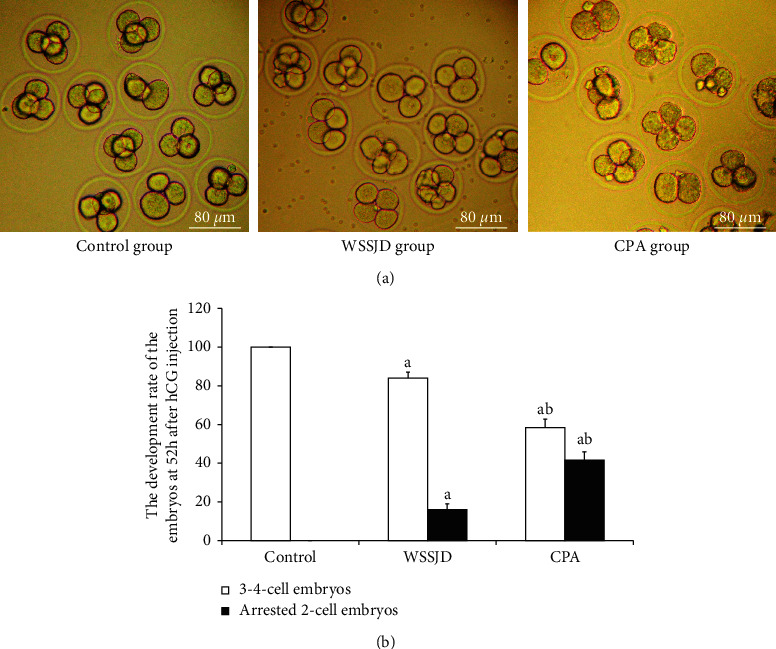
Embryos obtained from the oviduct of mice at 52 h after hCG injection. (a) The 3-4 cell stage embryos were obtained from the oviduct of the mouse 52 h after hCG injection. (b) The development rate of 3-4 cell stage embryos and the developmental arrest rate of 2-cell stage embryos collected at 52 h after hCG injection in different experimental groups were, respectively, counted (*N* = 10). Compared with the control group, ^a^*P* < 0.05; compared with the WSSJD group, ^b^*P* < 0.05.

Embryos were collected from the mouse oviducts at 41-42 h, 43-44 h, 45-46 h, and 52 h after HCG injection. The collected embryos included pronuclear embryos, 2-cell embryos, 3-4 cell embryos, and fragmented embryos (Figures 4(a) and 5(a)). Moreover, development rate of embryos in each stage was calculated (Figures 4(b) and 5(b)). At 41 h-42 h after HCG injection, the ratio of pronuclear embryos in the WSSJD group was significantly higher than that in the control group (*P* < 0.05)and was significantly lower than that in the CPA group (*P* < 0.05) (Figure 4(b)). There was no significant difference in terms of the 2-cell embryo development rate between the WSSJD group and the control group. However, the development rate of 2-cell embryos of the WSSJD group and control group was significantly higher than that in the CPA group (*P* < 0.05). The rate of fragmented embryos in the CPA group was significantly higher than that in the WSSJD group and the control group (*P* < 0.05). At 43 h-44 h after HCG injection, the ratio of pronuclear embryos and fragmented embryos in the WSSJD group and the control group was significantly lower than that of the CPA group (*P* < 0.05), while the development rate of 2-cell embryos of the WSSJD group and the control group was significantly higher than that of the CPA group (*P* < 0.05). There was no significant difference in the pronuclear embryo development rate, the 2-cell embryo development rate and the rate of fragmented embryos between the WSSJD group and the control group. After 45 h-46 h after HCG injection, the pronuclear embryos all developed into 2-cell embryos or fragmented embryos, and no pronuclear embryos were collected. The ratio of 2-cell embryos and fragmented embryos in the WSSJD group was not significantly different from that of the control group. However, the development rate of 2-cell embryos in the WSSJD group was significantly higher than that in the CPA group, while the ratio of fragmented embryos was significantly lower CPA group. Thus, at 41–46 h after HCG injection, WSSJD promoted 2-cell embryo development and reduced embryo degeneration.

The embryos collected from the oviduct at 52 h after HCG injection were 2-cell embryos and 3-4 cell embryos (Figure 5(a)). Embryos collected from the control group all developed to 3-4 cells, while some embryos collected from the WSSJD group and CPA group were arrested at the 2-cell stage. WSSJD group had a significantly higher development rate of 3-4 cell embryos while a significantly lower developmental arrest rate of 2-cell embryos than the CPA Group (*P* < 0.05). Therefore, WSSJD alleviated the 2-cell embryonic developmental arrest caused by CPA.

### 3.5. Expression of H3K27me3 in 2-Cell Embryos with Developmental Arrest

**Figure 6:**
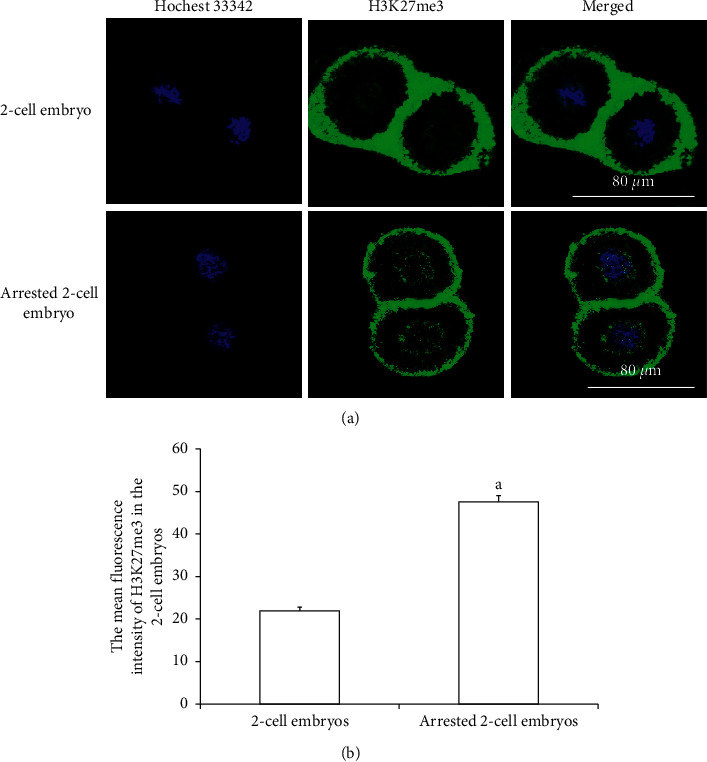
H3K27me3 expression in 2-cell embryos with developmental arrest. (a) Immunofluorescence labeling of H3K27me3 in normally developed 2-cell embryos and developmentally retarded 2-cell embryos. H3K27me3 is marked in green, and the embryonic nucleus is marked in blue. (b) The average fluorescence intensity of H3K27me3 in embryonic cell nuclei (*N* = 10). Compared with the normal 2-cell embryo group, ^a^*P* < 0.05.

H3K27me3 immunofluorescence staining was performed on 2-cell embryos with developmental arrest collected from both WSSJD and CPA groups at 52 h after HCG injection and the normal-developed 2-cell embryos of the control group (Figure 6(a)). The average fluorescence intensity of H3K27me3 in the embryos was calculated (Figure 6(b)). The results showed that the expression level of H3K27me3 in the 2-cell embryos with the developmental arrest was significantly higher than that in the normal developing 2-cell embryos (*P* < 0.05).

### 3.6. Expression of H3K27me3 Enzymes in Developmentally Arrested 2-Cell Embryos

**Figure 7:**
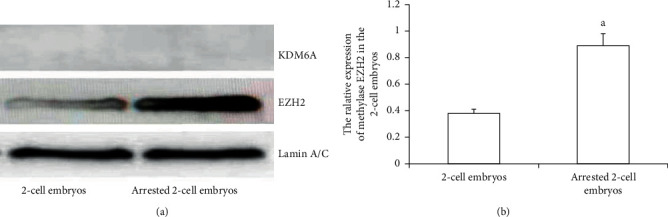
Expression of demethylase KDM6A and methyltransferase EZH2 in 2-cell embryos with developmental arrest. (a) Western blotting detected the expression of demethylase KDM6A and methyltransferase EZH2 in normal developing 2-cell embryos and 2-cell embryos with developmental arrest. (b) ImageJ image analysis software was used to analyze the relative expression level of EZH2 (*N* = 3). Compared with the normal 2-cell embryo group, ^a^*P* < 0.05.

The collected 2-cell embryos with developmental arrest from both WSSJD and CPA groups and normal 2-cell embryos in the control group were subjected to Western blotting analysis of demethylase KDM6A and methyltransferase EZH2 (Figure 7(a)). The expression of the demethylase KDM6A was not detected in the 2-cell embryos with normal development or 2-cell embryos with developmental arrest. However, the expression of the methyltransferase EZH2 was detected in both embryos. EZH2 expression was significantly higher in the arrested 2-cell embryos than that in the normally developed 2-cell embryos (Figure 7(b), *P* < 0.05). This result indicates that increased expression of EZH2 may improve the expression level of H3K27me3 in 2-cell embryos with developmental arrest.

### 3.7. Expression of Zygotic Genome Activated Genes in Developmentally Arrested 2-Cell Embryos

**Figure 8:**
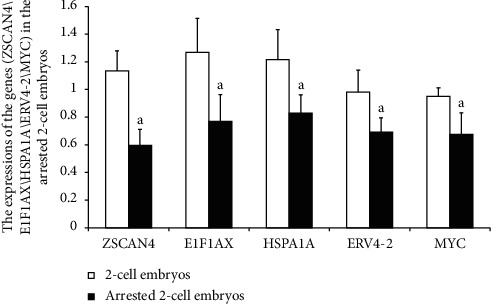
qRT-PCR analysis of the expression of zygotic genome activation genes (*ZSCAN4*, *E1F1AX*, *HSPA1A*, *ERV4-2*, and *MYC*) in 2-cell embryos with developmental arrest (*N* = 3). Compared with the normal 2-cell embryo group, ^a^*P* < 0.05.

The collected 2-cell embryos with developmental arrest from both WSSJD and CPA groups and normal 2-cell embryos from the control group were subjected to qRT-PCR analysis of zygotic genome activation genes (*ZSCAN4*, *E1F1AX*, *HSPA1A*, *ERV4-2*, and *MYC*) (Figure 8). The results showed that the expression of zygotic genome activation genes (*ZSCAN4*, *E1F1AX*, *HSPA1A*, *ERV4-2*, and *MYC*) in developmental arrest 2-cell embryos was significantly lower than that of the normally developed 2-cell embryos (*P* < 0.05). This suggests that the developmental arrest of 2-cell embryos may be related to the failure of zygotic genome activation.

## 4. Discussion

WSSJD inhibits the apoptosis of spermatogenic cells and sperm [2], improves the synthesis and secretion of testosterone [1], promotes sperm maturation, and improves semen quality [3], thereby exerting a beneficial effect on the maintenance of testicular function. It is widely used in the treatments of male spermatogenesis disorders. In this study, we demonstrated that WSSJD promoted the development of seminiferous epithelium, reduced the level of modification of H3K27me3 in sperms and male pronucleus, and the rate of developmental arrest of 2-cell embryos, and improved the development of early embryos from mice with spermatogenesis disorders induced by cyclophosphamide. H3K27me3 is a key epigenetic modification for transcriptional inhibition in sperms and embryonic cells. The developmental arrest of 2-cell embryos caused by CPA may be due to the increased expression of H3K27me3 methyltransferase EZH2 in embryonic cells, which increases the level of H3K27me3, inhibits the expression of zygotic genome activation genes, and leads to zygotic genome activation failure. This study provides evidence for the effect of WSSJD on the development of the early embryos after treating male spermatogenesis disorders.

It has been found that some environmental factors or drugs can cause changes in the modification pattern of H3K27me3 in sperm or embryos [26–28]. González-Rojo et al. found that 2000 *μ*g/L BPA (bisphenol A) increased the expression of DNA hypermethylase and H3K27me3 demethylase in testicular tissue, thereby interfering with the epigenetic modification of H3K27me3 in spermatogenic cells [26]. Santangeli et al. exposed female zebrafish of F0 generation to 20 *μ*g/L BPA, and the abundance of H3K27me3 in embryos of F1, F2, and F3 generations at 24 h after fertilization changed significantly, resulting in the silence of related gene expression [27]. Low-dose Chlordecone insecticide can significantly increase the modification level of H3K27me3 in fetal oocytes, change the epigenetic modification characteristics of oocytes, and lead to the delay of estrus, the reduction of the number of primordial follicles, and the increase of the atretic follicle in adult female mice [28]. Cyclophosphamide (CPA) is an alkylating agent that is widely used in antitumor and immunosuppression after organ transplantation [29–32]. It has been reported that the use of CPA in males significantly increased the rate of embryo loss, malformation, and the incidence of behavioral defects in offspring, and these abnormalities could be passed on to offspring [29]. However, the specific mechanism of action on sperm and early embryos is still unclear. In this study, we found that CPA abnormally increased the expression of the histone methyltransferase EZH2 and the modification level of H3K27me3 in sperm or male pronucleus, causing a 2-cell embryo developmental arrest. This implies that the developmental arrest of 2-cell embryos may be related to the abnormal expression of H3K27me3. The modification pattern of H3K27me3 in sperm or early embryo at a sensitive stage of development is very susceptible to some substances or drugs [26–28].

Failure to activate the zygotic genome is considered the main cause of 2-cell embryonic developmental arrest in mice [33, 34]. It has been found that zygotic genome activation is regulated by H3K27me3, which affects the expression of zygotic genome activation genes (ZSCAN4, E1F1AX, HSPA1A, ERV4-2, and MYC), leading to the developmental arrest of 2-cell embryonic development in mice [35–37]. In this study, we found that the expression level of H3K27me3 was significantly increased in 2-cell embryos with developmental arrest caused by CPA, while the expression level of zygotic genome activation genes (ZSCAN4, E1F1AX, HSPA1A, ERV4-2, and MYC) was significantly reduced. Therefore, it is speculated that the high level of H3K27me3 modification caused by CPA may inhibit the expression of zygotic genome activation genes (ZSCAN4, E1F1AX, HSPA1A, ERV4-2, and MYC) and cause the failure of zygotic genome activation, which may serve as the main cause of developmental arrest in mouse 2-cell embryos.

HPLC analysis of the active ingredients in WSSJD found that the active ingredients of WSSJD included ginsenoside, icariin, lividazine, and velvet antler polypeptide, etc. (data not published). Although these active ingredients can participate in the epigenetic regulation of different types of human cells [7–10], their effects on epigenetic regulation of sperms and early embryonic cells have not been reported. In this study, we found that WSSJD could significantly maintain the low level of H3K27me3 modification in sperms and male pronucleus of spermatogenesis impaired mice, increase the development rate of early embryos, and reduce the developmental arrest of 2-cell embryos. Therefore, we speculate that H3K27me3 modification may be an important target of WSSJD, but it is not yet known which active ingredient in WSSJD regulates H3K27me3 modification in sperm or early embryos. Further studies are needed.

## 5. Conclusions

In summary, we found that WSSJD promoted the development of seminiferous epithelium, reduced the developmental arrest of 2-cell embryos, and increased the rate of embryonic development by maintaining a low level of H3K27me3 modification in sperm and male pronucleus of spermatogenesis impaired mice. This study may broaden our understanding of the treatment mechanism of WSSJD, and for the first time, emphasized its regulation of epigenetic modifications in sperms and early embryos.

## Figures and Tables

**Table 1:** The sequence of primers used in real-time PCR.

Gene	Primer sequences (5′ to 3′)	
	Forward	Reverse
*HSPA1A*	TGGTGCAGTCCGACATGAAG	GCTGAGAGTCGTTGAAGTAGGC
*MYC*	ATGCCCCTCAACGTGAACTTC	CGCAACATAGGATGGAGAGCA
*EIF1AX*	GGAGACTACTGTTCTGGGTAGC	GTTACCGAGAGATCAAACACCG
*ERV4*	GGAGACTACTGTTCTGGGTAGC	GTTACCGAGAGATCAAACACCG
*ZSCAN4*	CCATGAGATCATACACATGCCAG	CAGTCAGATCTGTGGTAATTCCTC
*GAPDH*	ATTTGGCCGTATTGGGCG	TCTCGCTCCTGGAAGATGGT

Note: *HSPA1A*, recombinant heat shock 70 kDa protein 1A; *MYC*, myelocytomatosis oncogene homolog; *EIF1AX*, eukaryotic translation initiation factor 1AX; *ERV4*, endogenous retroviral sequence 4; and *ZSCAN4*, zinc finger and SCAN domain containing 4.

## Data Availability

The data used to support the findings of this study are available from the corresponding author upon request.
